# Genetic Risk Factors of Creutzfeldt-Jakob Disease in the Population of Newborns in Slovakia

**DOI:** 10.3390/pathogens10040435

**Published:** 2021-04-06

**Authors:** Dana Kosorinova, Girma Belay, Dana Zakova, Martin Stelzer, Eva Mitrova

**Affiliations:** Department of Prion Diseases, Faculty of Medicine, Slovak Medical University, 831 01 Bratislava, Slovakia; girma.belay@szu.sk (G.B.); dana.zakova@szu.sk (D.Z.); martin.stelzer@szu.sk (M.S.); eva.mitrova@szu.sk (E.M.)

**Keywords:** Creutzfeldt-Jakob disease, prion diseases, E200K, M129V, risk factors

## Abstract

The most frequent human prion disease is Creutzfeldt–Jakob disease (CJD). It occurs as sporadic (sCJD), genetic (gCJD), iatrogenic (iCJD) form and as variant CJD. The genetic form represents about 10–15% of confirmed cases worldwide, in Slovakia as much as 65–75%. Focal accumulation of gCJD was confirmed in Orava region. The most common point mutation of the prion protein gene (*PRNP)* is E200K. CJD has a long asymptomatic phase and it is not known when the carriers of the mutation E200K become infectious. Precautions to prevent iCJD are focused especially on clinical CJD cases, but asymptomatic CJD-specific mutation carriers cannot be excluded, and represent a potential genetic CJD-risk group. The aim of this study was to determine the occurrence, frequency and geographic distribution of the E200K mutation among the newborns, comparing the areas of focal accumulation of gCJD with extra-focal ones, as well as distribution of the polymorphism M129V of the *PRNP* gene. A total of 2915 samples of dry blood spots from anonymous newborns were analyzed. We used RealTime PCR method to determine the presence of the E200K mutation and the M129V polymorphism. Genetic testing revealed 13 carriers of the E200K mutation. Investigation of the M129V polymorphism affirmed higher representation of methionine homozygotes (48% MM, 44% MV, 8% VV). Achieved results fully confirmed our previous observations concerning both the specific and nonspecific genetic CJD risk among the Slovak general population. The 48% of methionine homozygotes and 4 carriers of the E200K mutation among 1000 live-born children in Slovakia underline the benefits of genetic testing.

## 1. Introduction

Prion diseases (Pd) are transmissible, fatal and so far incurable neurodegenerative diseases affecting humans and animals. The most frequent human Pd is Creutzfeldt–Jakob disease (CJD). CJD occurs in three forms: sporadic (sCJD) whose origin is unknown; genetic (gCJD), caused by specific mutations of the prion protein gene (*PRNP*); and iatrogenic (iCJD); which is acquired by therapeutic or invasive diagnostic measures. While in most of countries the genetic form represents about 10–15% of all CJD cases, in Slovakia it is characterized by considerably higher incidence of gCJD (65–75% of all confirmed cases), mainly in the north of Slovakia, in the Orava region [1]. There are about 40 mutations that have been described in the *PRNP* gene. The most common mutation associated with gCJD is a point mutation E200K (gCJD^E200K^) and in Slovakia it represents almost all gCJD cases [2], except the cases with R208H mutation [3] and P238S (not published yet). It influences formation and clinical course of the disease. This mutation was detected in 35% of healthy bloodline relatives of gCJD^E200K^ and its penetrance is about 59% in Slovakia [4]. Creutzfeldt–Jakob disease has a long asymptomatic phase, but data are lacking about when carriers of the mutation in the course of preclinical period become infectious. They all are considered as a “genetic CJD-risk group” in the population. According to our knowledge, data on the occurrence and frequency of asymptomatic carriers of the mutation E200K are not available.

The increasing trend of gCJD^E200K^ occurrence in Slovakia (1990 yr.—0.75/mil., 2018 yr.—2.75/mil.), caused by one type of point mutation, predominance of methionine homozygotes among the CJD patients (78.6%) [4] and in the general population as the absence of any data on the prevalence of the mutation E200K, accelerated our efforts for missing information concerning the mutation associated with the fatal and at present incurable disease.

Our study focused on the prevalence of the E200K mutation started by the investigation of corneal donors [5]. Later, we included into our analysis DNA from two clinical studies, but all three tested groups had a serious disadvantage, since we were not able to influence the number and the geographical distribution of tested samples. This important condition was finally fulfilled by testing anonymized DNA samples of newborns from all Slovak regions with increased (focal) and low (extra-focal) occurrence of gCJD^E200K^.

Preliminary partial results achieved from anonymous DNA samples of 1. newborns and 2. patients of two other clinical studies have been published [6]. They demonstrated asymptomatic carriers of the mutation E200K in persons without known relationship to gCJD affected relatives, not only in gCJD^E200K^ clusters but even outside of the focal accumulation of the disease.

The aim of the present study was to confirm their validity by 1. analyzing larger number of samples in a homogenous cohort, 2. focusing on the geographic distribution of specific (E200K) and nonspecific (polymorphism M129V) genetic risk factors in Slovakia, including the comparison of focal with extra-focal areas of gCJD.

## 2. Methods

We obtained 2915 samples of dry blood spots (on FTA cards) from anonymous newborn donors from Slovak Newborn Screening Center. There were 1389 born in 2007 (2.55% of all live-born children in Slovakia) and 1526 born in 2012 (2.74%). DNA was isolated by paramagnetic particles method (ZyGem Corporation Ltd., Hamilton, New Zealand) and subsequently the presence of the point mutation of the E200K on the *PRNP* gene was determined by Real Time PCR using TaqMan hybridization probes (Applied Biosystems, Foster City, CA, USA). Simultaneously, 588 DNA samples were examined by the same method in order to detect the M129V polymorphism.

## 3. Results

Genetic testing revealed 13 carriers of the E200K mutation, 9 from the Orava region, 1 from the central region and 3 from the eastern Slovakia region (Table 1). DNA analysis confirmed occurrence of the CJD–specific mutation carriers in the population of Slovakia in subjects with any known relationship to a patient with gCJD. The findings correlate with the incidence of genetic CJD in Slovakia and despite the fact that the tested Orava cohort was significantly smaller than the number of samples from the other Slovak regions, asymptomatic carriers of the mutation E200K in the gCJD^E200K^ focal area significantly exceeded (*p* = 0.00089789) the findings in extra-focal ones.

## 4. Discussion

The penetrance of the E200K mutation in Slovakia is incomplete, and at present we are not able to differentiate between the carriers that develop the disease. However, it could not be excluded that even asymptomatic “healthy” carriers of the mutation may be a source of the infection and represent a risk of iatrogenic CJD. This possibility is supported by experiments where animals without any symptoms of prion disease show high levels of infectivity [7].

Slovakia, similarly to some other countries, is also characterized by another increased genetic risk. The polymorphism M129V of the *PRNP* gene was confirmed as a nonspecific genetic risk factor for all transmissible spongioform encephalopathies, (influencing the susceptibility to CJD and the outcome of the disease) and also for other neurodegenerative diseases [8]. Methionine homozygosity (Met/Met) increases risk of iatrogenic [9], sporadic [8], genetic [4] and most significantly, of CJD variant [10]. The frequency of Met/Met at codon 129 is markedly different between the European (32–45%) and East Asian (92–94%) normal population [11,12,13,14,15]. This genotype is the most represented in Slovakia (48% MM, 43% MV, 9% VV) [5]. Our investigation of the M129V polymorphism on 588 anonymous individuals from different regions of Slovakia confirmed the previous data on higher representation of methionine homozygotes (48% MM, 44% MV, 8% VV). These data point to a relatively high nonspecific genetic CJD risk, that is, an increased susceptibility to the disease.

**Figure 1:**
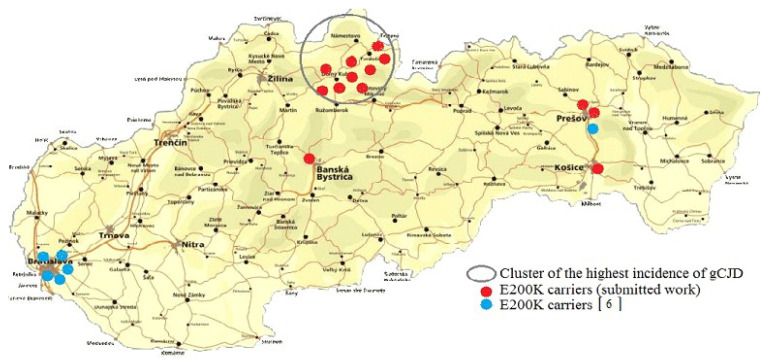
Total findings of the E200K mutation in subjects without any known relationship to Creutzfeldt-Jakob disease (CJD) affected in population of Slovakia.

In the presented study, 2915 newborns without any known relation to gCJD^E200K^ patients were tested; 13 asymptomatic carriers of the CJD-specific mutation E200K were identified. Nine of them were from gCJD^E200K^ focus; four were from the extra-focal areas (Figure 1). Achieved results fully confirmed our previous observations concerning both the specific and nonspecific genetic CJD risks in the Slovak general population [6]. From the prospective point of view, considering the penetrance of the disease in Slovakia, obtained results indicate approximately eight clinically manifested gCJD^E200K^ in the future. Examination of DNA samples of newborns from years 2007 and 2012 provide the first evidence of four carriers of the E200K mutation among 1000 live-born children in Slovakia. Described data may not be without interest mainly to other countries with an increased occurrence of gCJD^E200K^ (Hungary, Italy, Israel, Chile).

Our repeatedly verified, increased specific (E200K) and nonspecific (M129V) genetic risk of gCJD^E200K^ in the Slovak general population highlights the benefits of genetic testing. The possibility to identify the mutation carriers by years or decades before the clinical onset is a specific feature of the genetic form of CJD. It provides a unique opportunity to reduce the risk of iatrogenic infection and creates an exceptional prerequisite for prophylaxis, early diagnosis and treatment, as soon as this would be available.

## Figures and Tables

**Table 1:** The occurrence of E200K mutation in newborns in different regions of Slovakia.

Region	DNA Tested (N)	E200K −	E200K +
Western Slovakia	811	811	**0**
Orava	728	719	**9**
Central Slovakia	745	744	**1**
Eastern Slovakia	631	628	**3**
Total	2915	2902	**13**

## Data Availability

The data presented in this study are available on request from the corresponding author.
